# Innate Immune Signaling Induces Interleukin-7 Production from Salivary Gland Cells and Accelerates the Development of Primary Sjӧgren’s Syndrome in a Mouse Model

**DOI:** 10.1371/journal.pone.0077605

**Published:** 2013-10-17

**Authors:** Jun-O Jin, Yoshinori Shinohara, Qing Yu

**Affiliations:** 1 Department of Immunology and Infectious Disease, the Forsyth Institute, Cambridge, Massachusetts, United States of America; 2 Division of Oral Rehabilitation, Kyushu University, Fukuoka, Japan; University of Bergen, Norway

## Abstract

Elevated IL-7 in the target tissues is closely associated with multiple autoimmune disorders, including Sjögren’s syndrome (SS). We recently found that IL-7 plays an essential role in the development and onset of primary SS (pSS) in C57BL/6.NOD-*Aec1Aec2* mice, a well-defined mouse model of primary SS. However, environmental signals that cause excessive IL-7 production are not well-characterized. Innate immune signaling plays a critical role in shaping the adaptive immune responses including autoimmune responses. We and others have previously shown that innate immune signaling can induce IL-7 expression in lungs and intestines of C57BL/6 mice. In this study, we characterized the effects of poly I:C, a double-stranded RNA analog and toll-like receptor 3 agonist, on the induction of IL-7 expression in salivary glands and on pSS development. We showed that poly I:C administration to C57BL/6 mice rapidly induced IL-7 expression in the salivary glands in a type 1 IFN- and IFN-γ-dependent manner. Moreover, poly I:C-induced IL-7 contributed to the optimal up-regulation of CXCL9 in the salivary glands, which may subsequently promote recruitment of more IFN-γ-producing T cells. Repeated administration of poly I:C to C57BL/6.NOD-*Aec1Aec2* mice accelerated the development of SS-like exocrinopathy, and this effect was abolished by the blockade of IL-7 receptor signaling with a neutralizing antibody. Finally, poly I:C or a combination of IFN-α and IFN-γ induced IL-7 gene expression and protein production in a human salivary gland epithelial cell line. Hence, we demonstrate that IL-7 expression in the salivary gland cells can be induced by poly I:C and delineate a crucial mechanism by which innate immune signals facilitate the development of pSS, which is through induction of IL-7 in the target tissues.

## Introduction

Sjӧgren’s syndrome (SS) is a systemic autoimmune disease that primarily affects exocrine glands [1-3]. The characteristic pathological changes include lymphocytic infiltration of salivary and lacrimal glands and production of autoantibodies, leading to destruction and secretory dysfunction of these glands. SS affects about 2-4 million people in the US, with patients suffering from dry mouth, dry eyes, various systemic symtoms and a higer risk of developing B cell lymphoma [1-3]. SS can occur as primary SS (pSS) or secondary SS, the latter is associated with other connective tissue diseases [4,5]. Both autoreactive T cells and B cells are essential for the development of SS [2,4,6-8]. T helper (Th) 1-, Th2- and Th17-associated cytokines, including IL-12, IFN-γ, IL-4 and IL-17, all play indispendable pathogenic roles in the development and onset of this disease [9-14]. 

Interleukin-7 (IL-7) is a non-hematopoietic-derived cytokine that plays an essential role in supporting normal T cell development and homeostasis at physiological levels [15-17]. Excessive IL-7 activity has been shown to enhance effector T cell responses, preferentially Th1 and T cytotoxic (Tc) 1 responses, which are characterized by IFN-γ production [18-21]. Elevated IL-7 levels are associated with multiple autoimmune disorders [20,22] and *in vivo* loss-of-function studies demonstrate critical pathogenic roles of IL-7 in a variety of autoimmune diseases, including inflammatory bowel disease [23-25], rheumatoid arthritis [20,21], type-1 diabetes [17,26] and experimental autoimmune encephalomyelitis [18]. Similarly, pSS patients also have elevated IL-7 levels in the target organs and circulation [27]. Our recent study [28] showed that administration of exogenous IL-7 accelerates, whereas blockade of endogenous IL-7 inhibits the development and onset of pSS in C57BL/6.NOD-*Aec1Aec2* (B6.NOD-*Aec*) mice, a well-defined model of pSS [29,30]. Moreover, similar to the other autoimmune disorders, the promoting effects of IL-7 on pSS are underpinned by enhanced Th1 and Tc1 responses in the target organs [28]. 

The regulation of IL-7 gene expression by external signals in autoimmune disease settings is not well understood. Activation of various Toll-like receptors (TLRs), including TLR2, -3 and -4, induces IL-7 expression in hepatocytes in a type 1 IFN-dependent manner [31]. We recently reported that polyinosinic:polycytidylic acid (poly I:C), a synthetic analog of viral double-stranded RNA and agonist of TLR3, induces IL-7 production in lung tissues in a type 1 IFN- and IFN-γ-dependent fashion [32]. Interestingly, activation of innate immune system through TLRs by viruses is a likely environmental trigger of pSS pathogenesis, together with the genetic predisposition [2,33-36]. Administration of poly I:C has been shown to accelerate the onset of SS in New Zealand Black X New Zealand White F1 (NZB/WF1) mice, a mouse model of secondary SS [36]. Indeed, TLR3 expression is easily detected in salivary gland epithelial cells from pSS patients and HSG cells, a human salivary gland epithelial cell line [37-39]. Furthermore, *in vitro* poly I:C treatment directly up-regulates several chemokines and B cell-activating factor (BAFF) in salivary gland epithelial cells from pSS patients [37]. 

The present study is undertaken to test the hypothesis that poly I:C can induce IL-7 expression in salivary gland cells and promote the development of pSS in part through this mechanism. By employing both *in vivo* and *in vitro* experimental strategies, we demonstrate that poly I:C induces IL-7 expression in salivary gland cells in a type 1 IFN- and IFN-γ-dependent fashion. Furthermore, by using B6.NOD-*Aec* mice, we showed that poly I:C accelerates the development of pSS-like exocrinopathy in an IL-7-dependent manner. Hence, these findings supported our hypothesis and delineate an IL-7-dependent mechanism linking innate immune signaling and enhanced T cell autoimmune responses in salivary glands that facilitate the development of SS. 

## Results

### Poly I:C induces IL-7 expression in the salivary glands in a type 1 IFN- and IFN-γ-dependent fashion

**Figure 1:**
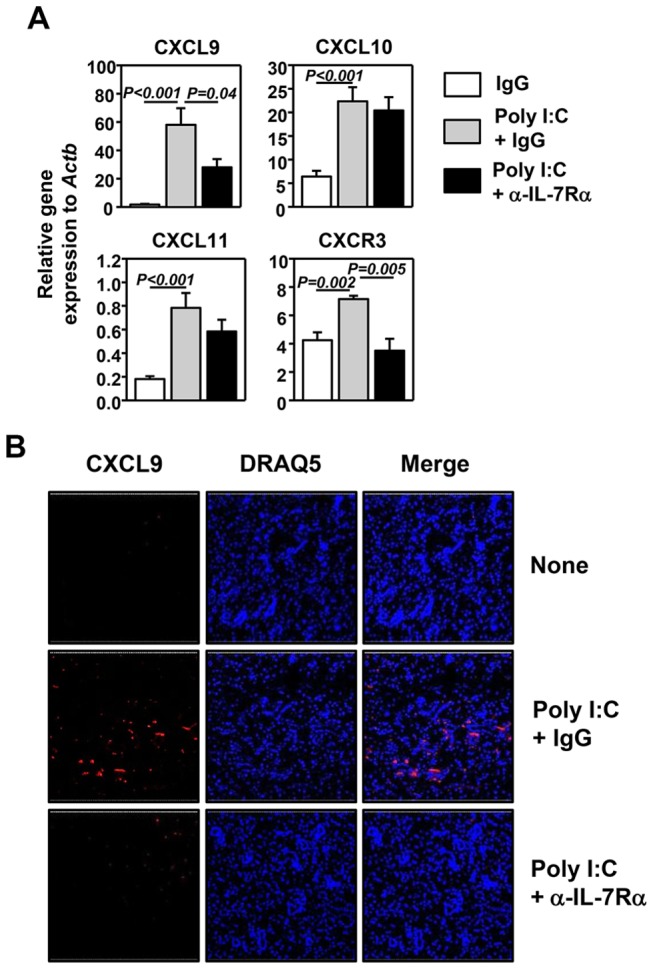
Poly I:C induces IL-7 expression in the submandibular glands. (A) Real-time PCR analysis of gene expression in submandibular glands from C57BL/6 mice 6 hours post poly I:C injection, presented relative to that of β-actin. Data are the average of analyses of 6 individual mice (3 mice per experiment, total 2 independent experiments). (B) Immunofluorescence staining of IL-7 in submandibular gland sections from C57BL/6 mice 24 hourse post poly I:C injection. Differential interference contrast (DIC) image of the same sample is also shown. Data are representative of analyses of 6 individual mice (3 mice per experiment, total 2 independent experiments). (C) C57BL/6 mice were pretreated with anti-IFNAR1 or anti-IFN-γ 2 hours prior to poly I:C injection. After 6 hours, relative IL-7 mRNA levels in lung tissue were measured by real-time RT-PCR. Data are from analyses of 6 individual mice (2 mice per experiment, total 3 independent experiments).

Our recent report showed that systemic injection of poly I:C induces lung inflammation and high levels of IL-7 production by lung epithelial cells [32]. We hence examined whether administration of poly I:C can induce similar events in the submandibular salivary glands, an important target site of SS. We injected 100 μg poly I:C intraperitoneally (*i.p.*) into C57BL/6 mice, and after 6 hours, measured mRNA levels of IL-7 in submandibular salivary glands. Poly I:C administration led to marked increase in IL-7 mRNA levels in the submandibular glands (Figure 1A) in addition to IFN-γIRF-7and IFN-α, known targets of poly I:C. Poly I:C treatment also increased IL-7 protein levels as determined by immunofluorescence staining (Figure 1B). To investigate whether poly I:C-induction of IL-7 in the submandibular glands is dependent on IFNs, we pretreated C57BL/6 mice with neutralizing anti-IFNAR1 or anti-IFN-γantibody before poly I:C administration. Interestingly, induction of IL-7 gene expression in the submandibular glands by poly I:C was almost completely inhibited by anti-IFNAR1 and was partially reduced by anti-IFN-γ (Figure 1C). These results suggest that poly I:C treatment is sufficient to induce IL-7 expression in the submandibular glands and its effects are dependent on IFN-α and IFN-γ. 

### Poly I:C-induced IL-7 contributes to the optimal up-regulation CXCL9 in salivary glands

**Figure 2:**
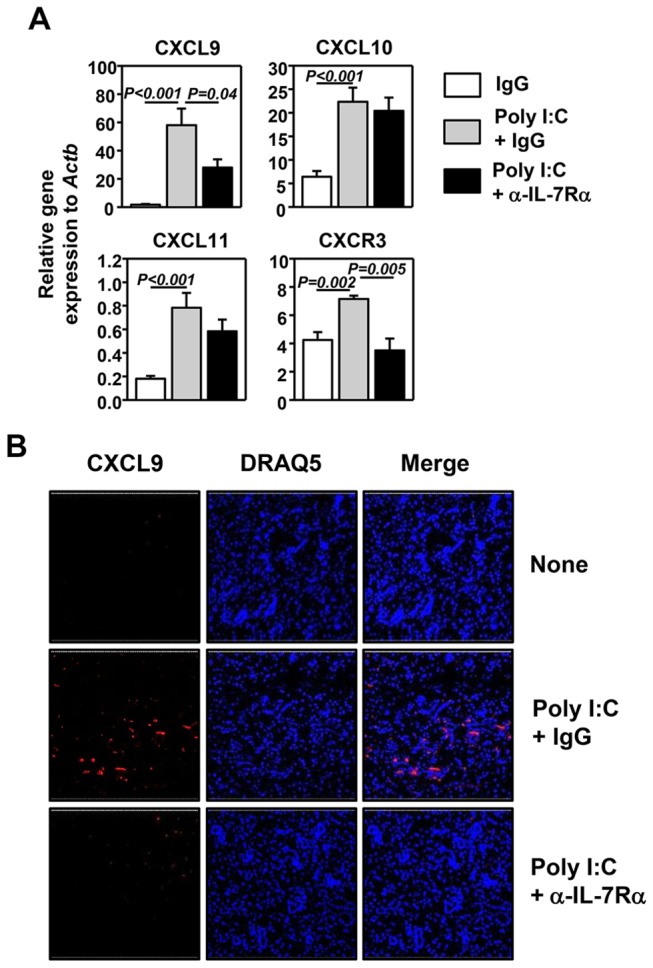
Poly I:C upregulates expression of CXCR3 ligands in submandibular glands in an IL-7-dependent fashion C57BL/6 mice were pre-treated with anti-IL-7Rα 2 hours prior to poly I:C injection. (A) Levels of CXCL9, 10, 11 and CXCR3 mRNA were measured from submandibular glands 24 hours after poly I:C administration, presented relative to that of β-actin. (B) Submandibular gland sections stained with CXCL9 and DRAQ5. All data are the average of or representative of 6 individual mice (2 mice per experiment, total 3 independent experiments).

We previously reported that poly I:C induces expression of all three CXCR3 ligands, CXCL9, -10 and -11, in the lung and that the induction of CXCL9 and -11 is dependent on IL-7 [32]. We therefore investigated whether poly I:C can similarly induce CXCR3 ligand expression in the submandibular glands through induction of IL-7. We treated C57BL/6 mice with poly I:C together with control IgG or anti-IL-7Rα, and 24 hours later, examined the mRNA levels of CXCR3 ligands in the submandibular glands. Poly I:C treatment induced a substantial increase in CXCL9, -10 and -11 mRNA levels (Figure 2A). Blockade of IL-7Rα significantly inhibited the induction of CXCL9 (P = 0.04), but did not affect that of CXCL10 and -11 (Figure 2A). Immunofluorescence staining showed that poly I:C treatment markedly increased CXCL9 protein levels in submandibular glands and this increase was completely abrogated by anti-IL-7Rα treatment (Figure 2B). Consequently, CXCR3, which is the receptor of CXCL9, -10 and -11 and expressed by IFN-γ-producing T cells and NK cells, was also increased by poly I:C treatment and this increase was completely abolished by anti-IL-7Rα (Figure 2A). Hence, IL-7 contributes to poly I:C-induced increase in CXCR3 and its ligand CXCL9 in the submandibular glands.

### NK cells mediate initial upregulation of CXCR3 ligands induced by poly I:C in the salivary glands

**Figure 3:**
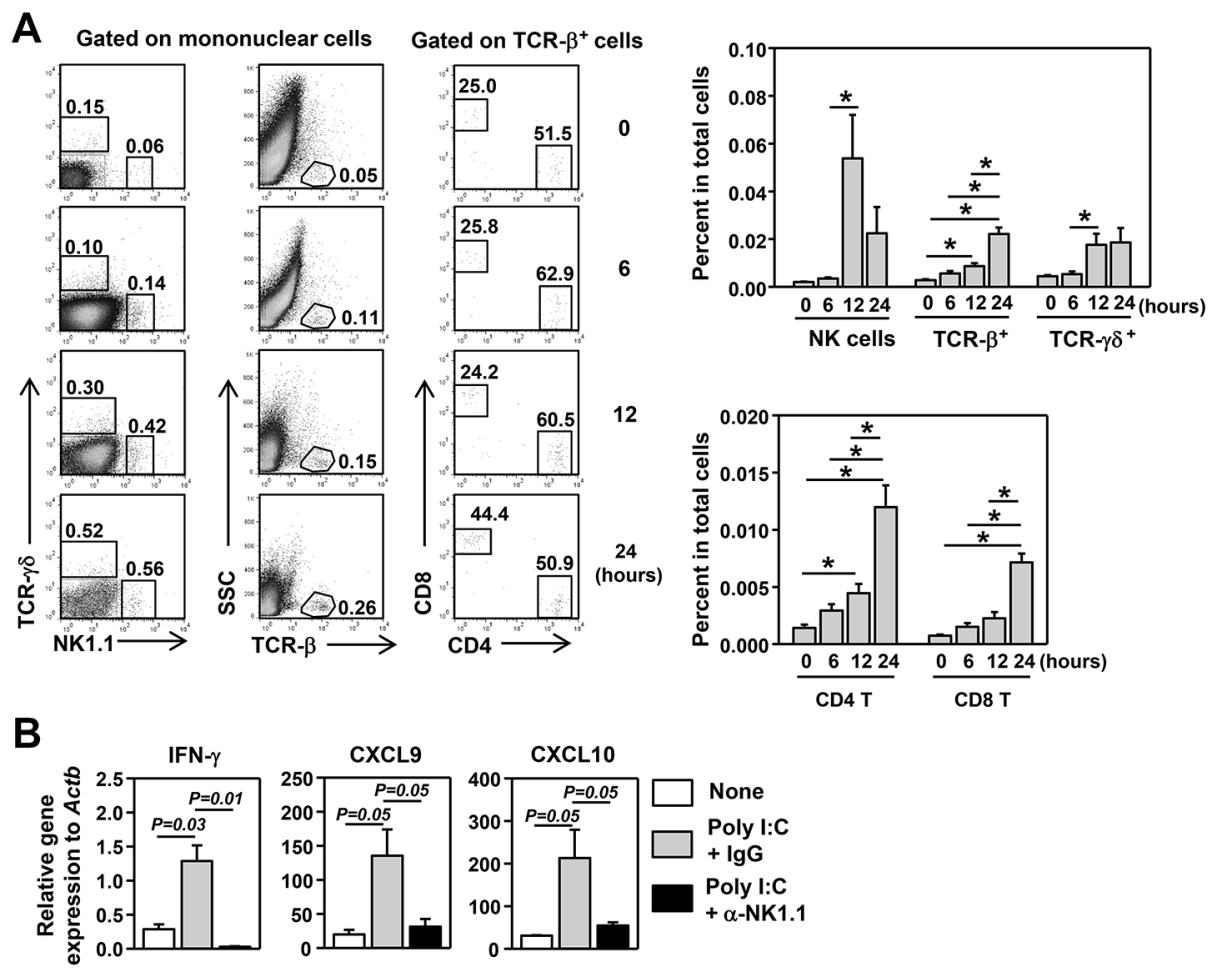
NK cells contribute to the early up-regulation of CXCR3 ligands induced by poly I:C. (A) C57BL/6 mice were treated with poly I:C by for the indicated amount of time. Left panels, surface staining for immune cell populations in the submandibular glands, with the gating indicated above the plots. Right panels, Percentage of NK1.1^+^ TCR-β^+^, TCR-γδ^+^, CD4^+^ and CD8^+^ T cells among total submandibular gland cells. All data are the average of or representative of 5 individual mice (2-3 mice per experiment, total 2 independent experiments). (B) RAG-1^-/-^ mice were pre-treated with anti-NK1.1 antibody 2 days prior to poly I:C injection. Submandibular glands were harvested 6 hours after poly I:C injection and measured for gene expression by real time PCR. Data are from analyses of 4 individual mice (2 mice per experiment, total 2 independent experiments).

We previously found that administration of exogenous IL-7 to C57BL/6 mice induces CXCR3 ligand expression in submandibular glands, and the effect of IL-7 requires T cell-derived IFN-γ [28]. We also showed that in lung tissues, poly I:C-induced IFN-γ from NK cells is crucial for the initial induction of CXCR3 ligands and influx of T cells and poly I:C-induced IL-7 is crucial for sustaining the CXCR3 ligand expression at later stage by increasing T cell-derived IFN-γ [32]. To examine whether the same mechanisms are operating in the submandibular glands, we first assessed the kinetics of immune cell infiltration in the submandibular glands after poly I:C treatment. The frequency of NK cells and TCR-γδ cells increased significantly within 12 hours post-treatment. Similarly, total TCR-β^+^ T cells and CD4 and CD8 T cells also increased at 12 hour post-poly I:C treatment (Figure 3A). To examine whether NK cells are the early sources of IFN-γ in response to poly I:C, we treated RAG1^-/-^ mice, which lack both T and B lymphocytes, with poly I:C and assessed expression of IFN-γ in the submandibular glands 6 hours later. Poly I:C induced up-regulation of both IFN-γ and CXCR3 ligands in RAG1^-/-^ mice in the absence of lymphocytes, but it could not do so when NK cells were depleted from these mice by treatment with an anti-NK1.1 antibody (Figure 3B). Hence, at early phase after poly I:C stimulation, the induction of IFN-γ and CXCR3 ligands in the submandibular glands is dependent on NK cells but not T cells. 

### Poly I:C treatment accelerates SS-like pathological changes in an IL-7-dependent manner in a mouse model of pSS

**Figure 4:**
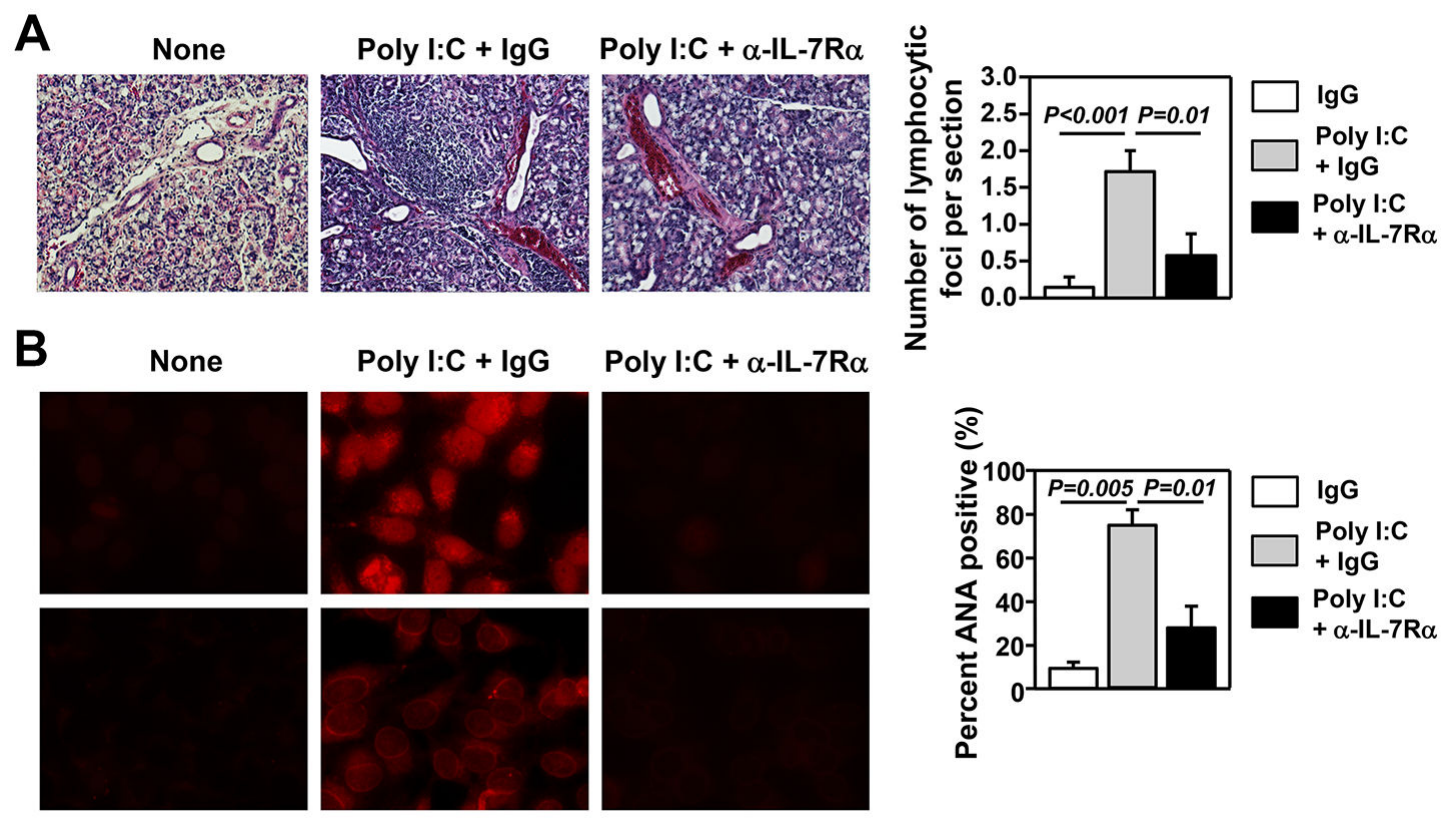
Poly I:C accelerates the development of SS in an IL-7-dependent manner. Poly I:C, together with either IgG or anti-IL-7Rα antibody, was administered to 12-week old B6.NOD-*Aec* mice 3 times weekly for 8 weeks. All the following parameters were measured after 8 weeks of consecutive injections. (A) H&E staining of submandibular gland sections (left panels) and focus score of leukocyte infiltration in submandibular glands (right panel). (B) Detection of serum ANA, left panels, and percentages of mice that are serum ANA positive, right panel. All data are representative or the average of analyses of 7 individual mice (1-3 mice per experiment, total 4 independent experiments).

It was reported that poly I:C accelerates the development of SS [36]. We therefore assessed whether the SS-accelerating effects of poly I:C are dependent on IL-7 by blocking IL-7R signaling with a non-depleting, receptor-blocking anti-IL-7Rα antibody. We *i.p.* injected 100 μg poly I:C plus anti-IL-7Rα or its isotype control IgG into B6.NOD-*Aec* mice 3 times weekly, starting from age 12 weeks. After 8 weeks of injection, we measured various disease parameters. Histological analysis showed increased amounts of leukocyte foci in the submandibular glands of poly I:C plus control IgG-treated mice, compared to non-poly I:C-treated mice (Figure 4A). In comparison, leukocyte infiltration was barely detectable in poly I:C plus anti-IL-7Rα-treated mice (Figure 4A). Analysis of serum antinuclear antibodies (ANA) against a human epithelial cell line, HEp-2, showed that poly I:C treatment markedly increased the percentage of mice that are serum ANA positive, compared to non-poly I:C-treated control mice, and this increase was abolished by IL-7Rα blockade (Figure 4B). Thus, poly I:C accelerates the development of salivary gland inflammation and ANA production in an IL-7-dependent fashion in a mouse model of pSS. 

### Poly I:C enhances Th1 and Tc1 responses in B6.NOD-Aec mice in an IL-7-dependent fashion

**Figure 5:**
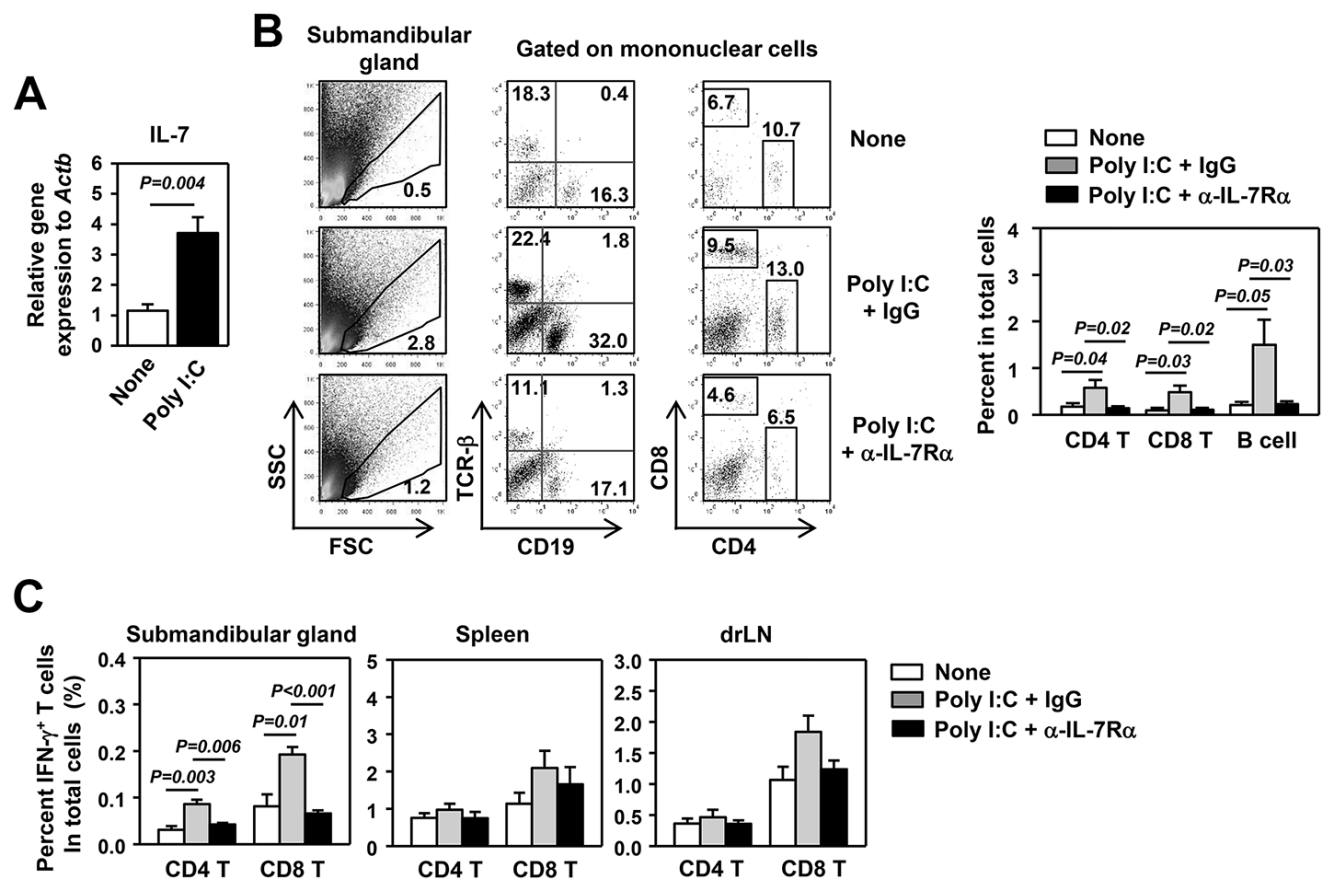
Poly I:C enhances Th1 and Tc1 responses in an IL-7-dependent manner. B6.NOD-*Aec* mice were injected with poly I:C plus IgG or anti-IL-7Rα antibody as described in Figure 4. (A) IL-7 mRNA levels in the submandibular glands of non-poly I:C- or poly I:C-treated B6.NOD-*Aec* mice. (B) Flow cytometric analyses of leukocyte subpopulations among mononuclear cells in submandibular glands. (C) Percentage of IFN-γ^+^ T cells in total submandibular gland cells, splenocytes and dr LN cells based on flow cytometric analysis. Data are representative of or the average of the analyses of 7 individual mice (1-3 mice per experiment, total 4 independent experiments).

Our recent studies showed that IL-7 promotes Th1 and Tc1 responses and plays a critical role in the development and onset of pSS in B6.NOD-*Aec* mice [28]. We therefore assessed whether poly I:C enhances Th1 and Tc1 responses in B6.NOD-*Aec* mice that have received poly I:C injection for 8 weeks. First we showed that, consistent with previous results, poly I:C up-regulated IL-7 mRNA levels in the submandibular glands compared to non-poly I:C-treated controls (Figure 5A). We next assessed the changes in lymphocyte populations. Poly I:C treatment markedly increased the percentage of mononuclear leukocytes, determined by forward- and side-scatter profiles, in the submandibular glands and this increase was abrogated by anti-IL-7Rα treatment (Figure 5B, left panels). Furthermore, poly I:C increased the percentage of CD19^+^ cells, TCR-β^+^ cells and CD4, CD8 T cells among submandibular gland-infiltrating mononuclear cells and their percentages among total submandibular gland cells, and these increases were abolished by anti-IL-7Rα treatment (Figure 5B right panels). Moreover, poly I:C treatment markedly increased the percentages of IFN-γ^+^ CD4 and CD8 T cells in submandibular glands, and these increases were abrogated by anti-IL-7Rα treatment (Figure 5C). In comparison, IFN-γ^+^ CD4 and CD8 T cells in drLN and spleen were not significantly affected by poly I:C treatment (Figure 5C), indicating that the Th1/Tc1-promoting effects of poly I:C are more prominent in the salivary glands than lymphoid organs. Hence, poly I:C enhances Th1 and Tc1 responses in salivary glands and accelerates the development of SS-like pathologies in an IL-7-dependent manner. 

### Poly I:C and IFNs induce IL-7 expression in human salivary gland epithelial cells

**Figure 6:**
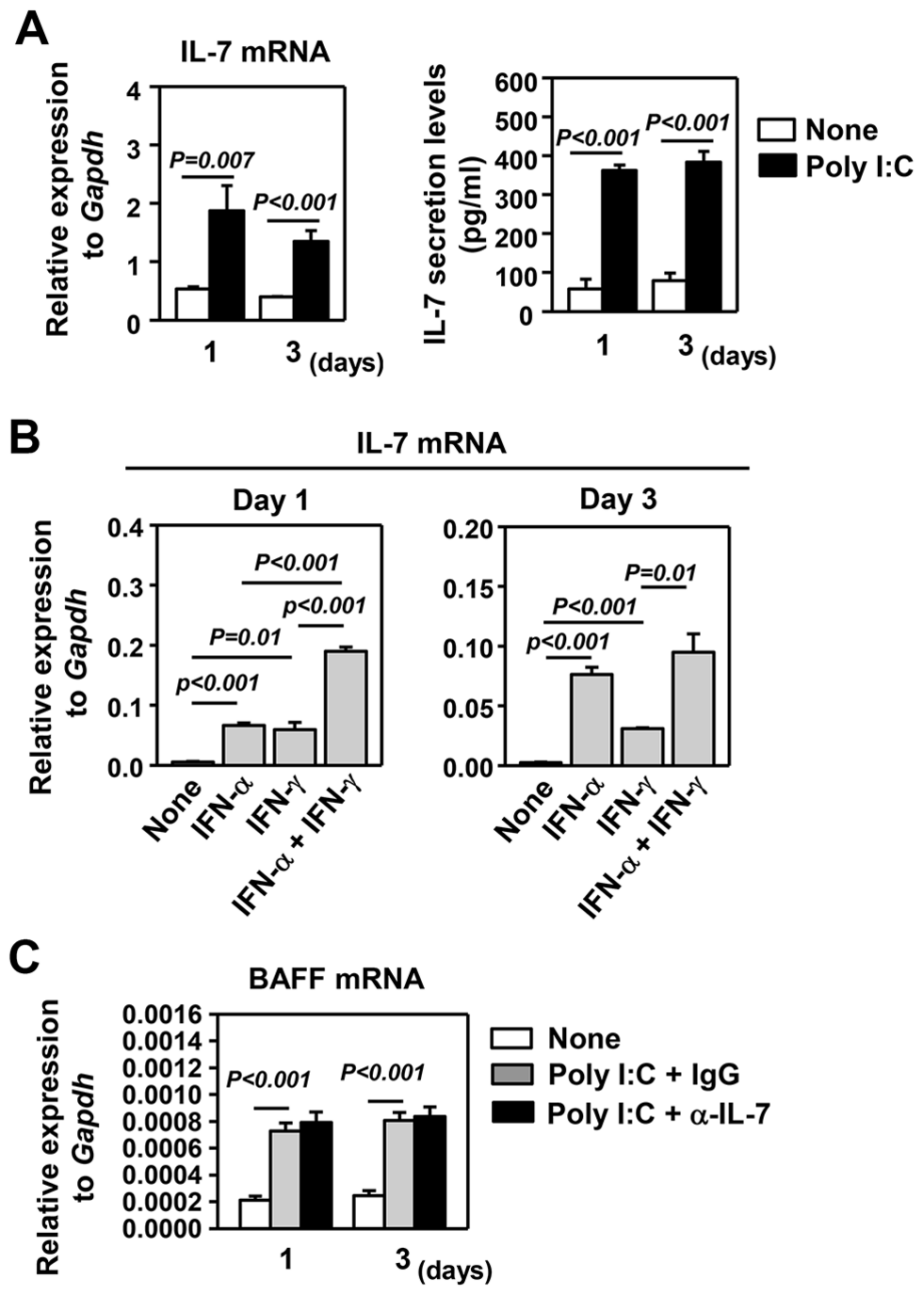
Poly I:C induces production of IL-7 in human salivary gland epithelial cells. (A) HSG cells were cultured *in* *vitro* with or without poly I:C for 1 and 3 days. IL-7 mRNA levels, left panel, and protein concentrations in culture supernatants, right panel, assessed by real time PCR and ELISA, respectively. (B) HSG cells were treated with IFN-α, IFN-γ or both for 1 and 3 days, and then measured for IL-7 mRNA levels. (C) HSG cells were treated with poly I:C in the presence of control IgG or anti-human IL-7 for 1 or 3 days, and then measured for BAFF mRNA expression. All PCR results are presented relative to that of GAPDH. All data are the average of analyses of 6 independent samples for each group (3 samples per experiment, total 2 independent experiments).

Having shown that *in vivo* administration of poly I:C induces IL-7 production in submandibular glands in both C57BL/6 and B6.NOD-*Aec* mice (Figure 1), we next directly determined the effect of poly I:C on the production of IL-7 by human salivary gland epithelial cells. We treated HSG cells, a human salivary gland adenocarcinoma cell line, with poly I:C *in vitro* and found that the treatment significantly increased IL-7 mRNA levels in HSG cells and its protein levels in the culture supernatants after 1 and 3 days of treatment (Figure 6A). Since poly I:C induces IL-7 expression in an type 1 IFN- and IFN-γ-dependent manner *in vivo* (Figure 1), we next determined whether IFN-α or IFN-γ is sufficient to induce IL-7 expression in HSG cells. IFN-α and IFN-γ each induced IL-7 mRNA expression at 1 and 3 day post-treatment and the combination of the two cytokines resulted in a synergistically enhanced effect (Figure 6B). Hence, poly I:C or the cooperation of IFN-α or IFN-γ can induce expression and production of IL-7 in HSG cells, confirming the findings obtained from *in vivo* studies. Finally, we examined whether the known BAFF-inducing effect of poly I:C on human salivary gland cells is dependent on the induction of IL-7. We treated HSG cells with poly I:C in the presence or absence of a neutralizing anti-human IL-7 antibody, and found that poly I:C induced BAFF mRNA expression at 1 and 3 day post-stimulation and this effect was not affected by the blockade of IL-7. Hence, the induction of BAFF by poly I:C is not dependent on IL-7. This is consistent with the notion that IL-7, produced by salivary gland epithelial cells, acts primarily on T cells but not salivary gland epithelial cells to exert its function. 

## Discussion

In the present study, we demonstrated that poly I:C-elicited innate immune signaling can induce IL-7 production from salivary gland cells and provide initial evidence that IFN-α and IFN-γ are necessary and sufficient in mediating this effect of poly I:C. Furthermore, we showed that poly I:C accelerates the development of SS-exocrinopathy in an IL-7-dependent fashion in B6.NOD-*Aec* mouse model of pSS. 

Excessive IL-7 production and activities in the target tissues are linked to multiple autoimmune disorders [20,22] and blockade or genetic ablation of IL-7 prevents or reverse these autoimmune diseases [17,18,20,21,23-26]. The signals and mechanisms that cause the excessive IL-7 production at target organs have remained largely uncharacterized. Here we provide evidence that innate immune signaling elicited by poly I:C stimulation can induce IL-7 gene expression and protein production in salivary gland epithelial cells, and the enhanced IL-7 production in turn promotes the development of SS-like pathologies by enhancing Th1 and Tc1 responses. It was previously reported that repeated poly I:C administration to NZB/WF1 mice, a model of secondary SS, accelerates the development of salivary gland inflammation characterized by dendritic, NK and CD4 T cell infiltration [36]. Here we showed that in a mouse model of pSS, poly I:C induces similar events. We also delineated an IL-7-dependent mechanism linking poly I:C-stimulation with enhanced CXCL9 expression and increased Th1 and Tc1 cells in the salivary glands. Apart from IL-7, type 1 IFNs and CXCR3 ligands, poly I:C has been reported to induce a panel of other cytokines and chemokines, such as TNF-α, IL-6 and BAFF, in salivary gland epithelial cells or antigen presenting cells [35,37]. Therefore, poly I:C likely employs multiple mechanisms to facilitate the development and onset of SS, with induction of IL-7 being one of the critical mechanisms. 

The signaling pathways that mediate poly I:C induction of IL-7 awaits further investigation. In addition to TLR3, poly I:C can also activate melanoma differentiation-associated gene 5 (MDA5) and RNA-activated protein kinase (PKR) pathways in a number of cell types, including lung epithelial cells [40-44]. In human salivary gland cells, poly I:C-induction of BAFF is completely dependent on PKR and partially dependent on TLR3 [44]. Thus, future studies will examine the requirement of PKR, TLR3 and MDA5 signaling pathways in poly I:C-induction of IL-7 in salivary gland cells. In addition, future studies will determine the *in vivo* effects of IFN-α and IFN-γ on the induction of IL-7 from salivary gland cells by administration of these cytokines to C57BL/6 and B6.NOD-*Aec* mice, and characterize the signaling and molecular mechanisms underlying their effects. In addition to TLR3, which was consistently detected at significantly amounts in salivary gland epithelial cells in SS patients, TLR2, -4 and -7 were also detected in these cells [37,39]. It is speculated that multiple types of viruses in one genetically susceptible individual may act in concert to trigger the development of SS. Hence, it will be important to characterize the effects of additional TLR signals and different types of viral infections on IL-7 expression in salivary gland cells. 

We found that the *in vivo* effects of poly I:C on IL-7 induction are, to a large degree, dependent on type 1 IFN and IFN-γ. In addition, either poly I:C or the combination of IFN-α and IFN-γ can induce significant levels of IL-7 production from a human salivary gland epithelial cell line. It was extensively reported that poly I:C induces IFN-α production from a variety of innate immune cells and non-immune tissue cells. The earliest sources of IFN-γ upon poly I:C stimulation are likely NK cells, as many reports have shown that poly I:C can rapidly induce IFN-γ production in NK cells [32,45-49]. Indeed, our data strongly suggest that NK-derived IFN-γ and T cell-derived IFN-γ are respectively responsible for early- versus late-phase CXCR3 ligand-induction and T cell infiltration after poly I:C treatment. At early phase, poly I:C induced a rapid increase in NK cells in salivary glands and induced up-regulation of IFN-γ, CXCL9 and -10 in an NK-dependent but T cell-independent fashion (Figure 3). The early induction of CXCL9 and -10 in turn cause the recruitment of initial T cells to the salivary gland tissues that can be observed after 6-12 hours of poly I:C treatment. At a later stage, once the first batch of T cells have migrated into salivary glands, poly I:C-induced IL-7 can subsequently promote the IFN-γ production from these T cells, thereby amplifying and sustaining CXCL9 expression and T cell infiltration. Supporting this notion, we previously showed that exogenous IL-7 administration rapidly up-regulates IFN-γ expression in salivary glands and lungs of C57BL/6 mice but not RAG1^-/-^ mice that lack T cells [28,32]. Therefore, IL-7 increases IFN-γ expression in salivary glands and lungs in a T cell-dependent manner, likely by enhancing Th1 and Tc1 responses. It is hence conceivable that, at the later stage after poly I:C treatment, poly I:C-induced IL-7 can act on salivary gland-infiltrating Th1 and Tc1 cells to enhance IFN-γ production, which in turn reinforces IL-7 production in salivary glands in a positive feed-forward manner. Hence, we propose a two-step model for the CXCR3 ligand-induction and T cell infiltration in salivary glands in response to poly I:C stimulation, with NK cells acting in the first, initiating step and IL-7 and T cells acting in the second, amplifying step.

The early induction of both CXCL9 and -10 by poly I:C requires NK cells. At later phase, the optimal and sustained expression of CXCL9 requires IL-7 but that of CXCL10 does not. Therefore, poly I:C-induction of CXCL10 is achieved via IL-7-independent mechanisms. It is known that the induction of CXCL9 expression is more strictly dependent on IFN-γ than that of CXCL10 and -11 [50,51], as the latter two can be induced by a wider array of signals, including type 1 IFNs and TNF-α. Therefore, although the differential requirements for IL-7 by CXCL9 and -10 awaits further investigation, we speculate that the sustained induction of CXCL9 by poly I:C requires IL-7-mediated sustained IFN-γ production from T cells, whereas that of CXCL10 can be achieved via other cytokines that are dependent on or produced by NK cells but are independent of IL-7. 

Studies in recent years have shown that elevated IL-7 levels correlate with the onset of many autoimmune diseases in human, and blockade of IL-7 in a number of mouse models abolishes or alleviates the diseases before or after disease onset. Our present study and a previous report [28] revealed critical pathogenic functions of IL-7 in B6.NOD-*Aec* mouse model of pSS. Our future investigations will assess whether IL-7Rα blockade can reverse or alleviate established SS after disease onset and whether local blockade of IL-7Rα in the exocrine glands can achieve similar outcome. Moreover, it will be important to definitively demonstrate that the pathogenic function of IL-7 in pSS is indeed dependent on Th1 and Tc1 responses, and to define the crucial signaling molecules and transcription factors that mediate the Th1/Tc1-promoting effects of IL-7, with STAT5, PI3 kinase, programmed death-1 and T-bet among the likely candidates. 

### Conclusions

We demonstrate that poly I:C-elicited innate immune signaling can cause excessive IL-7 production in the SS disease setting to enhance the subsequent T cell autoimmune responses and accelerate the development of pSS-like exocrinopathy. This information will enable future in-depth studies on the regulation of IL-7 production and its functions in pSS and other autoimmune disorders and will facilitate the development of new therapeutic strategies to combat these diseases by inhibiting IL-7 production and activity. 

## Materials and Methods

### Mice

C57BL/6 and RAG-1^-/-^ mice were purchased from the Jackson Laboratory and B6.NOD-*Aec* mice were from University of Florida. All mice were kept under pathogen-free conditions. All animal experiments were carried out according to the National Institutes of Health regulations and with the approval from the Institutional Animal Care and Use Committee at the Forsyth Institute, which is operating under the requirements of the Association for Assessment and Accreditation of Laboratory Animal Care.

### Antibodies and cytokines

Cells were stained and analyzed on a FACSAria III cell sorter (Becton Dickinson), with dead cells excluded by forward light scatter. The following fluorescence-conjugated antibodies were used: CD4 (GK1.5), CD8α (536-7), TCR-β (H57-597), NK1.1 (PK136) and IL-17 (TC11-18H10.1) and anti-mouse CXCL9 (MIG-2F5.5) were from BioLegend; CD19 (ebio1D3) and IFN-γ (XMG1.2) from eBioscience. Purified monoclonal rat-anti-mouse IL-7RαRisotype control rat-IgG2a (2A3) and rat-IgG1 (HRPN) were from BioXcell, NH; neutralizing anti-IFNAR-1 (MAR1-5A3), anti-IFN-γ (XMG1.2) and anti-human IL-7 (BVD10-40F6) were from BioLegend. Human recombinant IFN-α and IFN-γ were from R&D Systems. 

### *In* ** *vivo* administration of poly I:C and other antibodies

C57BL/6 mice were *i.p.* injected with 100 μg of poly I:C (Sigma-Aldrich, MO) and sacrificed at different time after poly I:C administration to harvest submandibular salivary glands for further analysis. In some experiments, C57BL/6 mice were *i.p.* injected with 100 μg of control IgG, anti-IFNAR1 or anti- IFN-γ 2 hours prior to poly I:C injection. Mice were sacrificed at different time after poly I:C administration for further analysis. For NK cell-depletion, C57BL/6 mice were *i.p.* injected with a depleting anti-NK1.1 antibody (200 μg) 2 days prior to poly I:C injection. 

### Administration of poly I:C and anti-IL-7Rα antibody to SS-prone mice

Female B6.NOD-*Aec* mice were *i.p.* injected with 100 μg poly I:C together with either 100 μg control IgG or anti- IL-7Rα antibody, 3 times weekly for 8 weeks, starting from age 12 weeks. Mice were then subjected to further analysis. 

### Histology and immunofluorescence staining

Tissue samples were fixed in 4 % paraformaldehyde, embedded in paraffin and sectioned to 5 μm thickness. Sections were then stained with hematoxylin and eosin (H&E) and examined for leukocytic infiltration. Number of leukocytic focus, which contains at least 50 leukocytes, in each salivary gland section was counted. For immunofluorescence staining, paraffin sections were subjected to deparaffinization, re-hydration and antigen retrieval. These sections were then incubated with goat anti-mouse IL-7 or PE-anti-CXCL9 (MIG-2F5.5) at 4°C overnight, followed by Alexa647-conjugated anti-goat-IgG. Some samples were further stained with DRAQ5 for 5 min. The samples were examined with a Leica laser scanning confocal microscope (Leica Microsystems). Images were average projections of three optical sections and processed with the Leica confocal software. 

### Preparation of single cell suspension

Submandibular salivary glands, submandibular lymph nodes or spleen were cut into small fragments and ground into tissue homogenates with frosted glass slides. Tissue homogenates were then filtered through a 100 μm nylon mesh, washed, and removed of erythrocytes with ACK lysing buffer. The single cells were resuspended in culture medium. 

### *Ex vivo* T cell stimulation and intracellular cytokine staining

Singles cell suspension prepared from various organs were stimulated *in vitro* with phorbol 12-myristate 13-acetate (50 ng/ml) and ionomycin (1 μM; both from Calbiochem) for 4 hours, with the addition of monensin (Biolegend, eBioscience) in the final 2 hours. Cells were then stained for surface markers and intracellular cytokines with the intracellular cytokine staining kit (Biolegend, eBioscience) following the manufacturers’ instructions. 

### Real-time RT-PCR

Total RNA was reverse-transcribed into cDNA using Oligo (dT) and M-MLV reverse transcriptase (Promega). The cDNA was subjected to real-time PCR amplification (Qiagen) for 40 cycles with annealing and extension temperature at 60°C, on a LightCycler 480 Real-Time PCR System (Roche). Primer sequences are: IL-7 forward (F), 5’-GGAACTGATAGTAATTGCCCG-3’ ; reverse (R), 5’-TTCAACTTGCGAGCAGCACG-3’, IFN-α F, 5’-ACCTCAGGAACAAGAGAGCC-3’; R, 5’-CTGCGGGAATCCAAAGTCCT-3’, IFN-β F, 5’-TAAGCAGCTCCAGCTCCAAG-3’; R, 5’-CCCTGTAGGTGAGGTTGATC-3’, IFN-γ F, 5’-GGATGCATTCATGAGTATTGC-3’; R, 5’-CTTTTCCGCTTCCTGAGG-3’, CXCL9 F, 5’-CCCTCAAAGACCTCAAACAGT-3’ ; R, 5’-AGCCGGATCTAGGCAGGTT-3’, CXCL10 F, 5’-CCAGTGAGAATGAGGGCCAT-3’ ; R, 5’-CCGGATTCAGACATCTCTGC-3’. Other sequences will be provided upon request. 

### ELISA

IL-7 concentration in supernatants from *in vitro* HSG cell cultures was determined using ELISA kits (Biolegend) according to the manufacturer’s protocols. 

### Detection of serum antinuclear antibodies (ANA)

ANA in mouse sera were detected using HEp-2 (human epithelial cell) substrate slides (INOVA Diagnostics). Briefly, fixed HEp-2 substrate slides were overlaid with 1:40 diluted mouse sera and incubated for 1 hour at room temperature in a humidified chamber. After PBS washes, the substrate slides were incubated with 1:100 diluted Alexa Fluor 568-conjugated goat-anti-mouse IgG (Invitrogen) for 1 hour at room temperature. After PBS washes, the slides were analyzed under a FSX100 fluorescence microscope (Olympus). All images were obtained with the FSX-BSW software with constant exposure of 0.1 second (Olympus). 

***Invitro* culture and treatment of HSG cells**. 0.1 X 10^6^ HSG cells were seeded in each well of a 6-well culture plate and incubated in the presence or absence of poly I:C (10 μg/ml), IFN-γ (10 ng/ml), IFN-α (10 ng/ml), poly I:C plus rat IgG1 (10 μg/ml) or poly I:C plus anti-human IL-7 (10 μg/ml) for 1 or 3 days. The cells and culture supernatants were then harvested for real-time RT-PCR and ELISA assays. 

### Statistical analysis

Statistical significance was determined by Student’s *t*-test (two-tailed, two-sample equal variance). P values equal to or smaller than 0.05 were considered as statistically significant. 
