# Correction: Innate Immune Signaling Induces Interleukin-7 Production from Salivary Gland Cells and Accelerates the Development of Primary Sjögren’s Syndrome in a Mouse Model

**DOI:** 10.1371/journal.pone.0108573

**Published:** 2014-09-16

**Authors:** The PLOS ONE Staff

Figure 1 is a duplicate of Figure 2. The authors have provided a corrected Figure 1 here.

**Figure 1:**
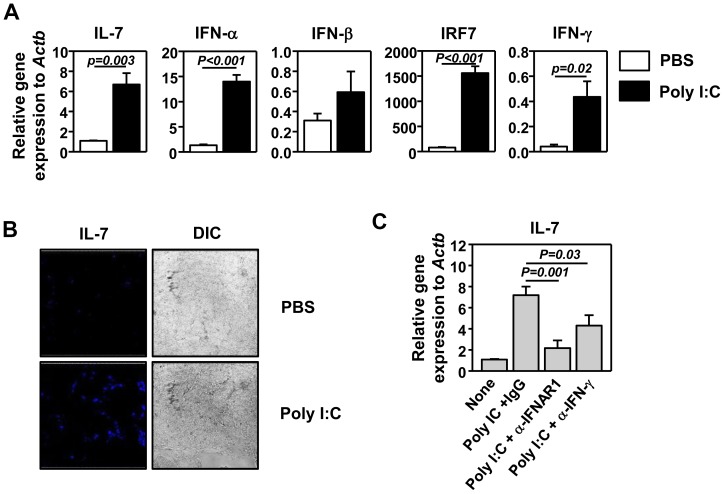
Poly I:C induces IL-7 expression in the submandibular glands. (A) Real-time PCR analysis of gene expression in submandibular glands from C57BL/6 mice 6 hours post poly I:C injection, presented relative to that of β-actin. Data are the average of analyses of 6 individual mice (3 mice per experiment, total 2 independent experiments). (B) Immunofluorescence staining of IL-7 in submandibular gland sections from C57BL/6 mice 24 hourse post poly I:C injection. Differential interference contrast (DIC) image of the same sample is also shown. Data are representative of analyses of 6 individual mice (3 mice per experiment, total 2 independent experiments). (C) C57BL/6 mice were pretreated with anti-IFNAR1 or anti-IFN-γ 2 hours prior to poly I:C injection. After 6 hours, relative IL-7 mRNA levels in lung tissue were measured by real-time RT-PCR. Data are from analyses of 6 individual mice (2 mice per experiment, total 3 independent experiments).
